# The Physical Learning Environment of Online Distance Learners in Higher Education – A Conceptual Model

**DOI:** 10.3389/fpsyg.2021.635117

**Published:** 2021-09-28

**Authors:** Cheuk Fan Ng

**Affiliations:** Centre for Social Sciences, Athabasca University, Alberta, AB, Canada

**Keywords:** physical learning environment, online learner, distance education, higher education, mobile learner

## Abstract

Online distance learning is offered not only in post-secondary distance education institutions but in traditional universities as well. With advances in mobile and wireless technologies, completing academic studies anywhere anytime should become feasible. Research in distance education and online learning has focused on computer-mediated communication, instructional design, learner characteristics, educational technology, and learning outcomes. However, little attention has been given to where exactly learners do their learning and studying and how the physical and social aspects of the physical environment within which the online learner is physically embedded (e.g., the home) supports and constrains learning activities. In this paper, the author proposes a conceptual model for understanding the role that the physical environment plays in online distance learning in higher education, drawing on theories and research in environmental psychology, online learning, telework and mobile work, and higher education. Several gaps in research are identified, and suggestions for future research are proposed.

## Introduction

Distance education has emerged as an important form of education in the last few decades (Lee, 2017; Xiao, 2018). Institutions, such as the British Open University and Canada’s Athabasca University, have offered university education online for some years. In recent years, traditional universities have begun offering online courses as well (Lee, 2017; Donovan et al., 2019). A recent national survey indicated that 30 percent of higher education students in the United States had taken at least one online course by distance (Ortagus, 2017). The popular use of portable and mobile devices in our daily lives and accessibility to wireless connectivity at home, workplaces, and many public places should make completing academic studies feasible in multiple settings, seemingly anywhere anytime and while on the move, as some have argued (Traxler, 2009; Hsu and Ching, 2015; Pimmer, 2016).

In the research literature on distance and online education, discussions have historically revolved around interactions between learner and content, other learners, teacher or facilitator (Moore, 1989), and the larger online community (Bozkurt et al., 2015). Until recently, little attention has been paid to one type of interaction: between learner and the physical environment. Regardless of what learning devices students use and what online instructional or learning environments they are in, students are embedded in the physical world (Graetz, 2006) and perhaps surrounded by people as well. The physical (e.g., ambience) and the social (e.g., alone or with others) contexts may support or hinder online learning activities.

There is a need to understand the complex relationships between learners, their ways of learning and studying, and the environments within which they study, both physical and virtual. The recent incorporation of information and communication technologies (ICT) on university campuses has led to investigations of such relationships within the facilities of traditional universities (e.g., Fisher and Newton, 2014; Beckers et al., 2015), but little research has focused on places beyond these campuses (e.g., students’ homes). Other studies have focused on informal learning in field settings (e.g., museum; Wang et al., 2017). With a few exceptions (e.g., Alphonse et al., 2019), research in online learning has not focused on where exactly learners do their learning and studying and how a physical place (e.g., the home) supports and constrains learning activities. Such an understanding would have implications for environmental designers, educators in pedagogical design, and online distance learners.

The purpose of this paper is to propose a conceptual model for addressing the role that the learner’s physical environment plays in online learning. The emphasis is on the physical environment though the virtual online environment is always in the background. The model is built upon literature in environmental psychology, online learning, telework and mobile work, and higher education. The focus is on learners pursuing formal university education at a distance in this digital age in developed countries. The paper will begin with an overview of online distance education, several relevant conceptual models, and then the proposed model. It is followed by a description of its components and the interrelationships between the components, and ending with a conclusion, suggestions for future research, and practical implications. My hope is for this paper to stimulate research into how pedagogical design of online distance education needs to consider the physical situated environment as well as its relations to the tools the learner uses. This seems particularly relevant during the current global pandemic (COVID-19) as educators need to teach online to students in diverse dwelling conditions and living arrangements, and access to computer devices and applications and internet connectivity.

## Online Distance Education

Historically, the goal of distance education was to provide post-secondary education to individuals, primarily adults who could not attend campus-based universities for personal, social, geographical, or other reasons (Lee, 2017). The delivery of distance education has evolved from the use of mail (correspondence courses) to analog audio-based (radio and audio cassette tape) and video-based (television and videotape) technologies, and later, to personal computers and the Internet (Lee and Chan, 2007). By using asynchronous and synchronous features, online learners can have control and flexibility in their learning regarding time and location (Shih et al., 2008). However, learning activities needed to be carried out at a specific physical location with a fixed device (Lee and Chan, 2007).

In recent years, the popular use of portable and mobile devices and accessibility to wireless technologies in our daily lives have stimulated a growing interest in the use of these technologies in higher education and distance education (Park, 2011). Apart from mobility and context, these technologies have the capability to incorporate multiple media (e.g., videos, text, and voice) and to facilitate “spontaneity, interactivity, informality, and ownership in learning” (Traxler, 2009). These capabilities have led to possibilities for developing multi-media, interactive course material, and learning activities to complete in multiple settings and while on-the-go. For example, students can use various functions on their mobile device (e.g., camera to take photos in the field) and share with other online learners, as in a graduate level graphic design course (Hsu and Ching, 2012).

Given these possibilities, Guri-Rosenblit (2009) has cautioned against perceiving online learning as the new generation of distance education; bridging over the digital divide and delivering cost-effective distance education in the digital age remain a challenge. The delivery of distance education in a cost-effective manner depends on economy of scale (Xiao, 2018). In addition, even though students in online distance education have become more diverse since the mid-1990s (Lee, 2017), it is those students who are older (Johnson, 2015) or who cannot afford campus-based education that are more likely to take online courses and programs (Ortagus, 2017).

Current developments in context-aware, situated learning could possibly be incorporated in online distance learning. Context-aware learning involves students accessing or be presented with information that are relevant to the physical location when the student is physically at that location (Hsu and Ching, 2015) and perhaps with augmented reality layers as well (e.g., Chang et al., 2013; Ryokai and Agogino, 2013).

## Relevant Conceptual Models

Next, several conceptual models that are relevant to online learners’ physical learning environments are described briefly. The Task Model of Mobile Learning and the models of telework and mobile work address how learners and knowledge workers, respectively, carry out cognitive tasks and communicate with others at one or more physical settings *via* the Internet. The Behavior Setting theory focuses on user behaviors and the social rules and norms within a specific physical setting, and the Task Model of Mobile Learning touches upon the physical context of the learner. Table 1 shows a comparison of these models.

**Table 1:** A comparison of models relevant to online distance learning in higher education (Authors).

Model (Authors)	People	Activities	Physical settings	Social context	Key ideas
Task model of mobile learning (Taylor et al., 2006)	Learners	Cognitive and communication	One of the contexts (independent, formalized, physical, and socialized)	One of the contexts (independent, formalized, physical, and socialized)	Three components of learning: learner, learning goals, and tools. Three components of mobile learning: context, control, and communication. Mobile technologies allow learners to achieve a learning goal in the most appropriate context *via* various communication channels and to control the learning process.
Model of telework (Standen et al., 1999)	Knowledge workers	Cognitive and communication	One (home) or two (home and organizational space)	Online and in-person	Variables in family/personal domain interact with variables in work domain to affect outcomes
Model of mobile work (Koroma et al., 2014)	Knowledge workers	Cognitive and communication	Multiple: home, organizational, public spaces	Online and in-person	Work outcomes affected by resources and barriers (physical, social, and virtual)
Behavior setting theory (Barker 1968; Wicker, 2002)	All	All user behaviors	One specific setting at a time	In-person	Physical milieu associated with a standing pattern of behavior. Furniture and equipment afford action possibilities. Social rules and norms support and constrain user behaviors

### The Task Model of Mobile Learning

Few existing models for designing mobile learning experiences and environments have focused on, or even mentioned, the physical environment as a component (review by Hsu and Ching, 2015). One of these few is Taylor et al.’s (2006) task model of mobile learning. This model comprises three basic elements of learning (i.e., learner, learning goal, and tools) and three essential components of mobile learning (i.e., context, control, and communication). The use of mobile technologies allows the learner to learn in an environment or context that is most appropriate and to control the learning process as well (Frohberg et al., 2009). For example, the learner’s current environment may be independent or having no relationship to the context of learning (i.e., learning from anywhere). On the other hand, the physical context could be relevant to the learning at hand at a particular time (e.g., during a field trip). How tools (e.g., mobile devices) are used would depend on the cognitive rigor. Control can range from tight teacher control to full learner control. Mobile technologies can improve communication and interaction by offering different communication channels. The scale of communication can vary from the isolated learner at one end to collaboration between teams at the other end (Frohberg et al., 2009).

### Models of Telework and Mobile Work

In telework and mobile work, the employee’s workspace is embedded within a physical setting or multiple settings. The physical environment is considered an essential component in conceptual models of telework and mobile work. For example, Standen et al.’s (1999) model of teleworking from home emphasizes how variables in the family or personal domain (dwelling size, household size and composition, activity pattern, and social support) and the work domain (social and physical work environment, job characteristics, and organizational characteristics) interact to affect job satisfaction, performance, and wellbeing. Similarly, effective mobile working is influenced by the resources and barriers present in multiple work settings. In Koroma, Hyrkkanen, and Vartiainen’s conceptual model (2014), they have identified several physical hindrances when working in multiple settings (e.g., limited working space) as well as associated challenges presented by the social environment (limited privacy and lacking social support) and the virtual environment (e.g., limited connections and access, and lacking ICT support).

### Ecological Theory

Ecological theories (e.g., ecological model of development; Bronfenbrenner, 1979) take a multi-level, systems approach to understanding how people’s behavior and wellbeing are influenced by their everyday surroundings and how people actively change their surroundings. The individual plays a role in the center of each context, and there is a transactional relationship between the individual and the context. The contexts are connected through a system of meso-system links.

Barker (1968) posited the concept of behavior setting as consisting of the physical milieu of a setting together with a naturally occurring, standing pattern of behavior within that setting. The traditional behavior settings include the home, schools, workplaces, coffee shops, and others. Within a behavior setting, affordances (Gibson, 1979), referring to properties in the environment that can provide functional possibilities for an individual as that individual sees it, are present (Heft, 2012). For example, a sofa at a public library affords sitting down to read a book. Each behavior setting has its furniture and equipment, and the participants’ behaviors within the behavior setting are regulated by social rules and norms. Participants’ choices are constrained, with the range of appropriate behaviors being maintained and inappropriate behaviors being sanctioned by the collective actions of other participants (Wicker, 2002; Heft, 2012). For example, a learner may use the dining table at home to do studying but would need to clear the table when supper time comes. Over time, the behavior setting will change in response to input from outside or actions by individual participants of the setting (Wicker, 2002).

Stokols (2018) has recently highlighted the influence of virtual features of our surroundings on our behaviors and wellbeing and how the cyberspace has had an important impact on the structure and functions of our built and social environments. The cybersphere has become intertwined with the built, natural, and social-cultural features of our environments, and contextual influences can be identified along spatial, temporal, sociocultural, and virtual dimensions.

From an ecological perspective, the online distance learner is at the center of each context that the learner is in (e.g., home, educational, work, and virtual). As the learner moves between locations, the transactional relationship between the learner and the context changes (Terras and Ramsay, 2012).

## Proposed Conceptual Framework

**Figure 1:**
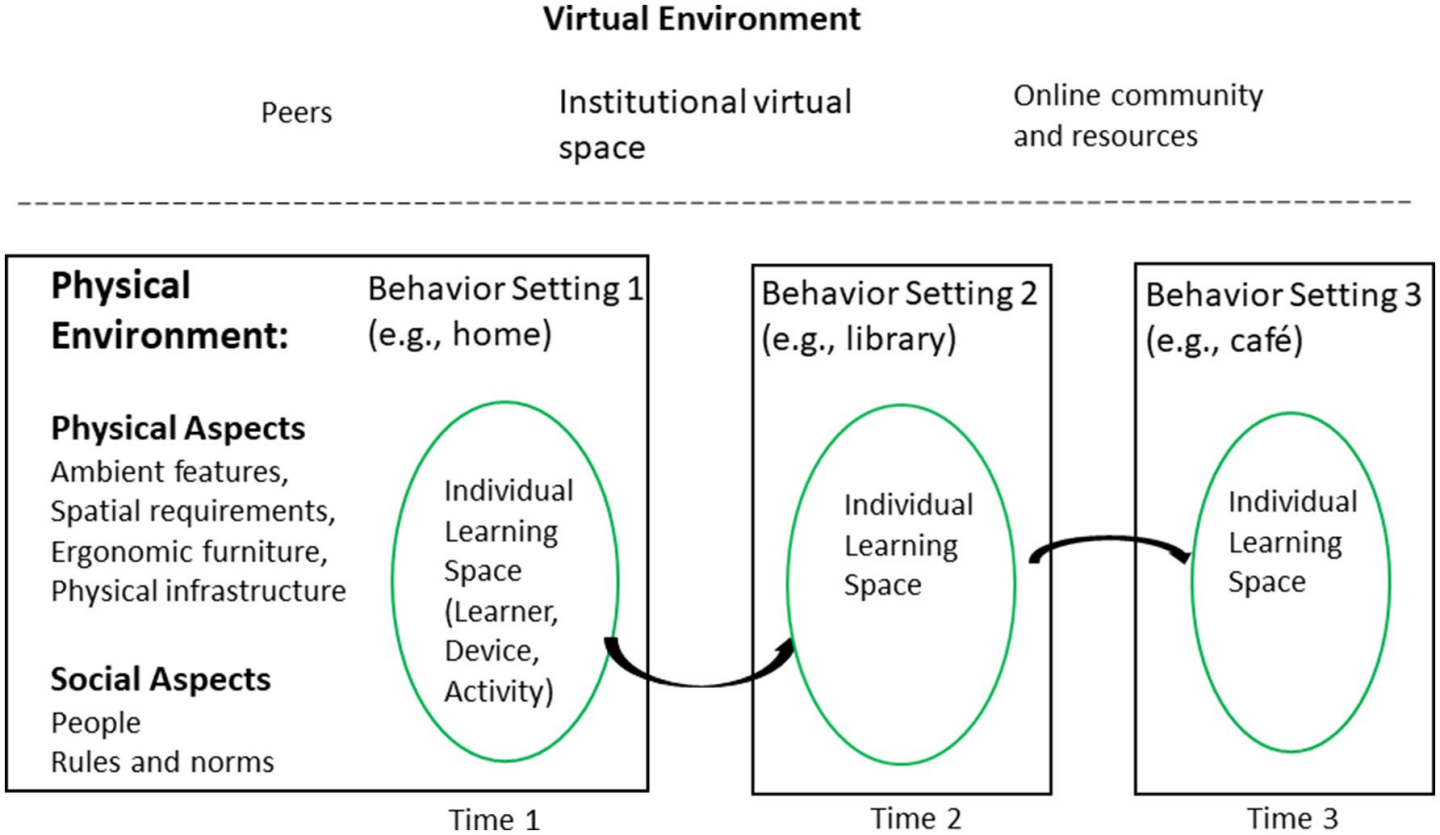
A conceptual model for understanding the role of the physical environment in online distance learning in higher education.

Building on the models outline above, I proposed a conceptual model that has three components: (1) a learner’s individual learning space (consisting of the learner, learning activities, and learning devices), (2) the physical environment (behavior setting) in which the learner is located, and (3) the virtual online environment (see Figure 1).

At any one time, an online learner’s individual learning space is embedded within one of several traditional behavior settings (e.g., home, library, workplace, café, and public transport). The learner carries out a number of learning activities (e.g., writing an essay, communicating with instructor, and taking photos) using technological devices that the learner has (e.g., desktop PC) or carries with him or her (e.g., smart phone) and furniture and equipment provided within that setting (e.g., a desk at home, a small table at a cafe, and wireless connectivity). The learning activities can be supported or hindered by the physical and social aspects of that behavior setting. At the same time, the learner is connected to the learner’s institutional virtual learning space, peers, and online community and resources, which can also provide support or present obstacles for the performance of learning activities. Over time, if and when the learner moves from one behavior setting to the next, a new set of activities, and supports and constraints may take over. The learner has the capacity to connect to the virtual environment *via* the Internet.

The following section describes each component of the proposed model, and the next section describes the relationships between these components.

### Behavior Setting

Unlike campus-based university students, online distance learners perform their learning activities in one or multiple behavior settings that are not necessarily designed as learning spaces (Figure 1). In several studies, working adult learners completing courses, programs, or work-based learning online at a distance reported that they studied mostly at home (Willging and Johnson, 2004; Nie et al., 2011; Selwyn, 2011; Alphonse et al., 2019). However, the workplace (Haythornthwaite and Kazmer, 2002; Nie et al., 2011) and public spaces (e.g., libraries, cafes, hotel rooms, airports, and buses) were used as well (Willging and Johnson, 2004; Nie et al., 2011; Bayne et al., 2013; Alphonse et al., 2019). The choice of setting(s) may have been influenced by the learner’s age, employment status, and program level, as most of these studies involved working adults in graduate programs.

In some cases, the behavioral setting itself is crucial for learning. Field trips or field work has been considered essential learning activities in some academic disciplines (e.g., geology and ecology). Depending on the learning goal, online learning activities could be designed to be carried out at local physical settings (e.g., museum) with context-aware mobile devices and applications that can detect the current context of the learner (e.g., location and time) and allow the learner to interact with the surroundings or to receive information pertinent to that particular context and time (Brown and Mbati, 2015; human geography field course, Jarvis et al., 2016).

Next, how the physical and social aspects of behavior settings can influence the learning activities of online learners are discussed. The physical environment includes the sensory stimuli from the built environment (e.g., lighting, noise, and temperature) and the physical presence of other people. The physical environmental can affect learning and performance through cognitive (attentional distraction and reduced concentration), physiological (temperature changes and comfort level), and affective means (e.g., motivation; refer to revised cognitive load model, Choi et al., 2014), especially when the learner is in a physical setting that is not primarily designed for learning (Terras and Ramsay, 2012). Empirical research has clearly shown that environmental stimuli from the physical learning environment can increase the cognitive load on learners’ working memory. As it takes effort for a learner to process irrelevant environmental stimuli, extraneous environmental stimuli should always be removed, or at the least, minimized (Choi et al., 2014).

When learners move from one physical setting to the next, they are exposed to many environmental cues, and changes in environmental stimuli can disrupt the engagement of the learner. Therefore, mobile learners need to develop skills in attention control to inhibit responses to extraneous stimuli (Terras and Ramsay, 2012). On the other hand, mobility from one place to another can be a resource for creativity through the provision of stimulation from different environments, people, and events, as in nomadic freelance creative work (Liegel, 2014) or as a relief from monotony experienced by mobile workers (Hampton and Gupta, 2008).

#### Physical Aspects

##### Ambient Features

The need for a functional and comfortable space (with control of temperature, noise, lighting, air quality, and ergonomic furniture) has been expressed by working adults in online graduate programs (Willging and Johnson, 2004; Alphonse et al., 2019), as with teleworkers (Montreuil and Lippel, 2003) and campus-based university students (Solvbert and Rismark, 2012; Beckers et al., 2016) who chose to work or study sometimes from home. Noise, lighting, and movement particularly can affect the learning of online learners.

Noise can impair an individual’s concentration and performance of complex tasks (Banbury and Berry, 2005). Meaningful background conversations (van de Poll and Sörqvist, 2016) and intermittent, unpredictable, or uncontrollable noise (Smith, 1985) are particularly detrimental. As with teleworkers (Gurstein, 1996) and mobile knowledge workers (Hislop, 2012), online graduate students have indicated their need for quietness at home or elsewhere to engage in individual, cognitive work (Alphonse et al., 2019). Some preferred studying with some continuous background sound (Scannell et al., 2016). Others use their iPod and headphones to block out prohibitive ambient noise or listen to their own music while studying. Engaging in any audio-rich online learning activities (e.g., language learning) can be difficult in a noisy environment without the use of headphones.

For reading and other viewing activities, online graduate students studying at home (Alphonse et al., 2019) have reported the need for adequate lighting and a preference for window access to view outside, as with teleworkers (Gurstein, 1996) and mobile knowledge workers (Brown and O'Hara, 2003). The lighting quality for computer work is dependent on several factors, such as illuminance, luminance, direction of light, glare, light source, screen design, and users’ visual ability (c.f. review by Osterhaus et al., 2015). Improper lighting, visual display position, and viewing distance contribute to “the computer vision syndrome” (eye strain, dryness, and neck and shoulder pain). The increased use of hand-held devices (e.g., e-readers and smart phones) under varying lighting conditions and closer viewing distances than desktop displays can present additional visual challenges (Gowrisankaran and Sheedy, 2015). Good display quality of computer tablets has been shown to cause less visual fatigue than poor display quality ones during long periods of viewing (Chen et al., 2016).

Studying with mobile devices while on the move is possible but can be challenging. To save time, graduate students completing online, work-based learning programs have reported using e-readers while travelling in public transport (Nie et al., 2011). The ambient conditions can constrain certain work activities when traveling in a vehicle, as reported by mobile knowledge workers (e.g., Hislop, 2012). Learning *via* listening to podcasts is possible while the learner is physically moving (walking or jogging), but learning is less effective than when sitting (Coens et al., 2011). Text input performance was reduced when the mobile device user was walking (Musić and Murray-Smith, 2016). Nevertheless, moving about within a physical setting and exploring with the help of a mobile device is itself part of situated learning (e.g., geography course; Jarvis et al., 2016).

##### Ergonomic Furniture

The computer workstation should be set up to allow the learner to sit directly in front of the computer screen with the top of the screen near eye level and the keyboard and mouse at elbow level. The chair should be height-adjustable and provides support to the user’s back (Honan, 2015). When working with a laptop, the user can make few adjustments to the body position, leading to neck and back pain and stress for the eyes and wrists (Janneck et al., 2018). Therefore, prolonged use of laptop would be better supported by an external monitor, mouse, and keyboard (Honan, 2015). Handheld devices, such as tablets and smartphones, can lead to wrist and neck pain if used for a long period of time. It is even worse working with a portable or mobile device on non-ergonomic furniture (e.g., at the kitchen table or on a sofa in the living room; Janneck et al., 2018).

##### Spatial Requirement

Researchers have reported that having a designated place was important for successful course completion online (Osborn, 2001; Holder, 2007; Alphonse et al., 2019) and telework (Hartig et al., 2007). Although some online learners live alone or have a study space set up, other adult distance learners reported having to set spatial boundaries between home and studies. They needed to negotiate a space within their household (Haythornthwaite and Kazmer, 2002; Selwyn, 2011) or with occupants of other spaces, as with teleworkers (Magee, 2000) and mobile workers (Hislop and Axtell, 2009). Men were more likely to have their own office, but women tended to study elsewhere within the home (Selwyn, 2011). With the use of portable and mobile devices, online learners should have the flexibility to move across locations within the home to complete learning activities, if they wanted. However, such mobility may be associated with the age of the learner. The working adult students in the Alphonse et al. (2019) study used only desktop computer and laptops, and wi-fi connections but not newer devices, and those in the Lee and Chan (2007) study preferred to listen to podcasts at a dedicated study location (typically at home) instead of listening to them on portable devices while doing other activities.

##### Physical Infrastructure

Online learners need to have access to high-speed Internet, wireless connection, power outlets, and a variety of computer devices, as do mobile knowledge workers (Hampton et al., 2010; Mark and Su, 2010) and campus-based students (e.g., Beckers et al., 2016). Today, most people in developed countries have high-speed Internet connections at their work, home, or school (Statistics Canada, 2021; Pew Research Centre, 2021a), and wi-fi has been commonly available in many public places (Doyle, 2011) for some years. However, data security remains a concern when working at coffee shops and other wi-fi hot-spots (Mark and Su, 2010). Cyber security can be a problem as well (Gaines, 2019).

Internet connection can be spotty and slow in those spaces with no wi-fi access (Seneca, 2014), and high-speed Internet services are less accessible to homes in rural areas in the United States (Pew Research Centre, 2021a). Low bandwidth restricts access to resource-rich materials (e.g., video-clips and video streaming) and the downloading of large files (Brown and Mbati, 2015). Cloud computing has now enabled online learners to store and access documents, audio, and video files *via* mobile devices from anywhere (Wang et al., 2014).

Persistent data services range from limited access to continuous access. Learners’ access is restricted by locations of wi-fi access points within a community or cellular network tower locations, or their cellular data services plan. Those learners with continuously available persistent network access can use many more functions (Grant, 2019). In contrast, mobile Internet access offers lower levels of functionality and content availability and operates on less open and flexible platforms (Napoli and Obar, 2014).

#### Social Aspects

How well a learner can engage in various learning activities can be affected by people’s activities, and the rules and norms of the behavior setting. Adult distance learners reported having difficulty not interacting socially with their family members, especially children, when at home (Haythornthwaite and Kazmer, 2002; Selwyn, 2011). Similarly, home teleworkers and mobile workers reported the need to negotiate rules regarding interruptions by people within and outside their home (Johnson et al., 2007) and in public places or transport (Hislop, 2012). Putting on the headphone to evade social interactions with those physically present and to communicate the need for privacy seems to have become a new social norm (Enriquez, 2013).

On the other hand, the presence of other people in the behavior setting could facilitate learning and studying in some situations, as social facilitation theory suggests (Zajonic, 1965). At the least, students reported high satisfaction when they watched videos for an online course together at the same location (Li et al., 2014).

Like teleworkers and mobile knowledge workers (Perry et al., 2001; Cooper and Kurland, 2002), students in distance education have reported feeling isolated (Wheeler, 2002). Building interpersonal relationships with others at home or in other behavior settings is important for learners pursuing academic studies completely online at a distance. The social support online learners received from family, friends, and colleagues have been reported to be an important predictor of student persistence (Holder, 2007; Ivankova and Stick, 2007; Lee and Choi, 2011).

### Individual Learning Space

Next, the second component of the proposed model, the individual learning space, comprising learning devices, the learner, and learning activities will be described. These are the three basic, inter-related elements of learning stipulated in the task model of mobile learning (Taylor et al., 2006). Likewise, the characteristics of the learner, learning task and its associated use of learning tools, and the interaction between learner and task characteristics are identified as the main factors affecting cognitive load and learning (Choi et al., 2014).

#### Learning Devices

Online learners must have access to appropriate learning devices and applications to be able to learn and study effectively at home and in multiple settings (Alphonse et al., 2019). To be effective, the applications need to be adapted to the tasks and the learner’s skill level, and technical assistance needs to be easily available to those students. Lack of technology preparation and technical support was identified as a reason for online learners to drop out of their programs (Willging and Johnson, 2004).

Learning devices may include desktop PC, portable devices (e.g., laptop), and mobile device (e.g., smartphones) for completing various learning activities in various physical settings. In a usability study, the users reported tablet PC to be less desirable than laptop PC, although the users could perform such tasks as reading well and were impressed by the general computing capabilities and portability of tablet PCs (Ozok et al., 2008). In another study, students found the iPad had enhanced their learning experience but not necessarily learning outcomes (Nguyen et al., 2015).

Ownership of mobile devices, such as smart phones, has increased rapidly in developed countries (Pew Research Centre, 2021b). In 2021, 85 percent of all adults in the United States owned a smart phone; of the 18–29-year-olds, 96 percent said they owned a smartphone (Pew Research Centre, 2021b). Mobile devices offer the benefits of portability, connectivity, convenience, expediency, immediacy, accessibility, individuality, and interactivity (Song, 2011, as cited in Terras and Ramsay, 2012). Mobile devices can support learning through enhancing users’ cognitive functions, such as performing calculations, note-taking, and accessing information *via* mobile internet (Terras and Ramsay, 2012). At the same time, these devices can also “solicit” demand for attention, as some proponents of the enactivism approach to cognition argue (Aagaard, 2018).

Usability for mobile phones is dependent on their features and physical limitations, technology, usage goals and environment, and user characteristics (e.g., compatibility between different platforms and devices, amount of human-device interaction, ergonomics, and readability and layout; Salazar et al., 2013). The small screen size of mobile devices can be problematic for users (Coursaris and Kim, 2011). A smartphone with larger screen (5.3 inches) was perceived more positively and easier to use than was a smartphone with smaller screen (3.7 inches; Kim and Sundar, 2014). Likewise, usability of mobile phone applications needs to be evaluated using standardized measurement scales (von Wangenheim et al., 2016) and for different devices and genres (Ahmed et al., 2018). Surprisingly, applications on phone platforms were perceived by users to be more usable than applications on the tablet platforms, partly due to ineffective mimicking of the large-screen functionality of desktop PC on tablet apps but effective focus of phone platforms on the core functionality needed by the users (Kortum and Sorber, 2015). The key is to use the right tool for the right job (e.g., smartphone for checking email and sending text updates, but larger-screen devices for extensive writing and other content creation activities; Honan, 2015).

Podcasts represent a low-threshold technology to deliver regular recordings of difficult, content-heavy material to learners who have little resources and a fear of technology (Gachago et al., 2016). Although some studies have reported students using mobile devices to listen to podcasts only infrequently when on the move (Lee and Chan, 2007; Evans, 2008; Pearce and Scutter, 2010), other studies have reported that students used e-readers or downloaded apps to read in short stretches of time while traveling or in public places, or when outdoors even with no access to an Internet connection (Nie et al., 2011; Seneca, 2014).

No doubt any technical limitations are temporary as advances in research in human-computer interactions are made to accommodate users’ physiological and psychological needs in response to ambient conditions and locations (e.g., home and train; Chen et al., 2008) and individual needs and preferences through various platforms, devices, and tools (e.g., Hsieh and Chen, 2016). For effective learning, mobile device should follow mobile design principles that are based on mobile user context, learning theory, and user interface design (Seneca, 2014), and learning applications design must consider the pedagogical effectiveness and technical functionalities and usability (Yau and Joy, 2010).

#### Learner Characteristics

Online learners today use technologies to different extents, and their skills and comfort levels vary (Gallardo-Echenique et al., 2015). Age could be a factor, as suggested in Alphonse et al.’s (2019) study of older, online graduate students. Whether or not “digital natives” have high digital literacy regarding the use of technologies for academic purposes is still being debated (cf. review by Gallardo-Echenique et al., 2015).

Social economic background continues to contribute to the digital divide (Guri-Rosenblit, 2009; van Deursen and van Dijk, 2018). Even within developed countries, such as the United States and Canada, those with lower income are less able to afford high-speed Internet services at home (Statistics Canada, 2021; Pew Research Centre, 2021a). In 2021, 15 percent of US adults were smartphone dependent (Pew Research Centre, 2021b), and the cost associated with cellular data plan is a legitimate concern (Grant, 2019). In the Netherlands, income level was associated with access to a diversity of devices and peripherals and the ability to afford maintenance costs for hardware, software, and subscription; such material access affected Internet skills, uses, and outcomes (van Deursen and van Dijk, 2018). Besides access, digital divide exists in psychological skills for appropriate use as well. Students’ usage of different technologies and their motivation may have different effects on academic performance. It is therefore important to provide training in information and digital literacy skills to support learners in their educational use of technology and to develop skills in screening out redundant or irrelevant input to their learning (Terras and Ramsay, 2012). More research is needed to examine other variables that are associated with students’ use of digital technologies in online learning.

#### Learning Activities

As with campus-based university students, online learners in higher education engage in various learning activities with their learning devices, broadly to include individual study of a cognitive nature and collaborative work with others that involve synchronous and asynchronous communication (Alphonse et al., 2019). Exploring (context-aware situated learning) and content creating (e.g., *via* wikis and microblogging) can be important activities as well (Terras and Ramsay, 2012).

##### Individual, Cognitive Work

When learning or studying at home, learners may be distracted or tempted to engage in other activities at the same time (campus-based students; Solvbert and Rismark, 2012). Such demands on attention may lead to a switching of attention from one task to another, or a sharing of attentional resources. When attention is divided between two tasks, performance is impaired, particularly when the two tasks are presented in the same sensory modality. When two tasks are performed close in time, performance of the primary activity can be affected negatively because of interference (cf. review by Levine et al., 2012).

To concentrate fully on individual cognitive work, online learners need to be free from interruptions and distractions when in a behavior setting. Interruptions can increase perceived workload and impair a learner’s performance of cognitive tasks (e.g., slowing the task down immediately after the interruption, van de Poll and Sörqvist, 2016; forgetting to carry out a task, Terras and Ramsay, 2012). It is harder for people to resume their original task when the interruption is long or there is little opportunity to rehearse the task goal during the interruption (Monk et al., 2008; review by Couffe and Michael, 2017).

Some physical learning environments have more distractions than do others. Several studies have shown that having a designated studying place that is relatively free from interruptions was a strong predictor of course completion for online learners (Osborn, 2001; Holder, 2007). Such designated space may be at home (Willging and Johnson, 2004). But for some online graduate students, it was difficult to have to manage family responsibilities while studying at home (Selwyn, 2011; Alphonse et al., 2019). Similarly, teleworkers and mobile workers have reported distractions from conflicting activities within and outside the home and in public places to be a challenge in maintaining focus (Johnson et al., 2007; Hislop, 2012).

##### Collaboration Through Oral Communication

To carry out collaborative learning activities orally online (e.g., on the phone, *via* Skype and Zoom), online learners need a quiet place to listen (if they do not want to wear headphones or ear buds) and talk. When talking in public spaces, they may have concerns about privacy for others physically present, as do mobile workers (Hislop, 2012). However, the social norm seems to be changing that it is becoming acceptable to talk to someone online in public while ignoring those physically present (Enriquez, 2013). Nevertheless, online learners can choose the communication channel that is most appropriate for a certain behavior setting. When there are barriers to communicating orally, the learner could communicate in text form even though this alternative form of communication may not be as effective.

### Virtual Environment

The third component of the proposed model is the virtual environment (Figure 1), consisting of the institutional virtual space (e.g., learning management system, institutional administration, course materials and resources, and instructors or facilitators), peers (i.e., fellow learners in the course or program), and other online communities and resources (e.g., community of practice and open educational resources).

### Relationship Between Individual Learning Space and Behavior Setting

As discussed earlier, the physical and social aspects of a behavior setting can support or hinder learning and studying. Further, the physical environment can interact with the task (including the learning device), the learner, or both to affect cognitive load and learning. For example, the effectiveness of instructional design and type of task is dependent on the characteristics of the physical environment, such as noise level. A learner’s skills level interacts with the ambient conditions of the physical environment to affect cognitive load and learning outcome (Choi et al., 2014).

For effective learning, learners need to be able to choose or control their physical learning environment. “Studying environment” has been shown to be significantly associated with academic performance, satisfaction, or course completion among online learners ranging from community college to graduate program level (Osborn, 2001; Holder, 2007). Managing physical environment has been identified as an important self-regulation skill for online distance learners (Kocdar et al., 2018). They need to develop skills to withstand the environmental interruptions while moving from location to location and to self-monitor and manage demands on their limited attentional resources (Terras and Ramsay, 2012).

The behavior setting itself affords learning. In situated learning, the physical context is relevant to the learning at hand at a certain time (Frohberg et al., 2009). Learning activities can be designed to be accomplished on site (e.g., a museum) and perhaps with the use of context-aware mobile devices and applications (Brown and Mbati, 2015; Jarvis et al., 2016). This can be particularly useful for skills training when the context in which learning takes place is similar as the context in which skills are tested (i.e., the context-dependent effect; Smith and Vela, 2001). Mobility between contexts may disrupt this supportive effect of context dependency because it is unlikely that memory encoding and recall will take place in the same context (Terras and Ramsay, 2012). If the goal is to facilitate transfer of learning, then learning should take place in various contexts (Choi et al., 2014).

### Virtual Environment and Its Relationship With Individual Learning Space

Learners with different characteristics use various portable and mobile devices and applications to carry out individual, cognitive learning activities, access resources, communicate with instructors, and interact and collaborate with peers *via* the Internet. In computer-supported collaborative learning, a variety of technical and digital tools and pedagogical strategies (e.g., discussion boards, simulations, and wikis) have been used to support learning and instruction that foster the social nature of learning (Sung et al., 2017; Jeong et al., 2019). Learners need to develop and maintain social networks and support with their peers online (Ivankova and Stick, 2007; Shackelford and Maxwell, 2012), and the online learning environment needs to be well-designed for fostering and enabling these social connections. There is a whole area of scholarship and research devoted to various components of the virtual environment and their relationships with learning, such as computer-mediated communication, learning community development, instructional design, and educational technologies that facilitate such activities and among learners with different characteristics (c.f. reviews of distance education research by Bozkurt et al., 2015; mobile learning research by Krull and Duart, 2017).

An emerging area of research concerns distraction and interruptions that result from using media to multitask while studying. So far, research has shown that using media devices to multitask during lectures (e.g., text messaging or checking Facebook) is common among college students (Moreno et al., 2012) and that multitasking has negative effects on academic performance (Rosen et al., 2013; Conard and Marsh, 2014; review by Levine et al., 2012). The extent of impairment depends on how similar (in modality in particular) and difficult the tasks are (cf. review by Chen and Yan, 2016). On the other hand, earlier studies have reported that most students did not engage in other activities while listening to podcasts (Lee and Chan, 2007; Evans, 2008; Pearce and Scutter, 2010), as the cognitive load of multi-tasking can be too much (Pearce and Scutter, 2010).

### Interrelationship Between Individual Learning Space, Physical Environment, and Virtual Environment

As in mobile working, online learning involves the learner using technologies to interface two environments – the immediate environment in which the learner is physically present and the virtual space of the learner’s institution or other learners – at the same time (Frohberg et al., 2009). Learners and instructors can choose, or are required, to access the virtual environments synchronously or asynchronously and perhaps from multiple physical locations. As with mobile knowledge workers (Mark and Su, 2010; Hislop, 2012), the mobile learner needs to be aware of what the distant instructor or the other learner is doing, where that person is, and what time it is to decide what types of access and interaction is possible or appropriate. For example, the teacher or student may be engaging in a separate activity or be interrupted by unrelated matters at his or her physical location while participating online (Jamieson et al., 2000). The learner can also consult with the online community and other online resources at the same time from where the learner is physically present. Learners can use social media to find out who is in close physical proximity and arrange to meet in person. At the same time, online messages and social intrusions can come from the virtual environment at any time, which may support or hinder learning activities.

Considering that learners can switch their psychological “presence” between the physical environment and the virtual environment, researchers could examine how learning effectiveness may be associated with congruence between the physical learning environment and the online environment (e.g., studying online in the library *versus* at home) in the future. When using mobile devices to learn in media-rich physical environments, information from the virtual environment may complement or compete with information from the physical space. For example, the combination of information sources may result in split-attention and redundancy effects, thus affecting students’ learning negatively (Liu et al., 2012). Mobile augmented reality, involving overlaying dynamic, location-based digital information on learners’ mobile devices (e.g., through videos), can allow learners to interact with and learn about the physical environment surrounding them. Although mobile augmented reality can keep learners more engaged, it can also direct attention away from the very environment they are learning about. In the end, how learners look at the environment is dependent on how information is presented to them by the instructor (Ryokai and Agogino, 2013). Such active engagement in learners is consistent with enactivist approaches to cognition, which emphasize the dynamic relations between brain, body, and environment (Gallagher, 2018).

### Time

Conceptually, the individual learning space can be considered a mobile space that moves from one behavior setting to the next over time (see Figure 1). The ability to manage time has been shown to be significantly associated with academic performance, satisfaction with the course, or course completion among online learners at various program levels (Osborn, 2001; Holder, 2007). As suggested in research in mobile work (Vartiainen, 2006), how long online learners stay in one setting and how frequently they move from one setting to the next could influence the effectiveness of learning. The optimal frequency and duration may depend on the extent to which course materials and learning activities are designed for fragmented learning.

One significant benefit of the Internet is to transcend geographical boundaries and time. Mitchell (2003), as cited in Fisher and Newton (2014), proposed a synchronous/asynchronous and virtual/physical matrix of learning opportunities: synchronous and local (face-to-face meeting); synchronous and remote (telephone, video conference, and text messages); asynchronous and local (site-specific signage and white board); and asynchronous and remote (internet web virtual studio; google). For synchronous activities, learners physically located in different parts of the world are to a great extent bound by time, which regulates their daily activities and the behavior settings they are in. Therefore, online social norm may dictate what kinds of communication and behaviors are appropriate and what are not (e.g., attending a skype meeting during nighttime).

### Flexibility and Control

Online students value flexibility and control in deciding what, where, and when to study (Nie et al., 2011). As with many teleworkers (Lundberg and Lindfors, 2002) and mobile knowledge workers (Hislop, 2012), online learners could move at various times between different settings that have different ambient features, interact with people, carrying out different tasks using the appropriate technology necessary for performing those learning activities (Solvbert and Rismark, 2012; Bayne et al., 2013). In practice, online learners have reported less flexibility in when and where studying can take place. Instead, many online learners established fixed routines of studying that were much influenced by their gender, life stage, and employment status; for example, some working adult learners made use of time during their commute to and from work and lunch breaks at work to study (Selwyn, 2011).

## Conclusion and Discussion

The physical environment plays an important role in online distance learning in higher education in this digital age. The physical environment that includes the physical infrastructure and space, and ambient features together with its social environment can support or hinder the performance of learning activities carried out by the learner with various computer and mobile devices. At the same time, the virtual learning environment is all encompassing, interacting with the learner’s individual learning space within a physical setting, As the learner moves from one physical setting to the next, the learner would encounter a new set of supports and barriers. The use of mobile technologies and devices facilitates such mobility, interactivity, and connectivity.

The proposed conceptual model provides a roadmap for future research that focuses either on elements of one of the three components: individual learning space, physical environment (behavior setting), and virtual environment, or on the interrelationships between the components.

For example, researchers may focus on the behavior setting component of the model in examining how physical learning spaces can be designed to support online learning. Empirical research that examines environmental opportunities for and constraints to learning and studying, and how learning takes place in typical behavior settings (even the home) is quite limited. Further research could examine how noise, lighting, other ambient features, ergonomics, and other variables in various behavior settings may affect the effectiveness and satisfaction of studying audio, visual, and multi-media online content. Also, how online learners set and negotiate spatial and social boundaries in various settings can be explored. As these behavior settings are dynamic in nature (Wicker, 2002), future research may explore how traditional behavior settings (e.g., café) are or will be transformed, replaced, or merged by actions taken by online learners. Other researchers may study how learners with different characteristics (e.g., personality and ability) prefer the use of different learning devices (currently available or yet to be developed) to achieve different learning goals (e.g., individual self-reflection or collaboration with others).

And yet other researchers may go beyond one component (e.g., individual learning space) to focus on its relationship with another component (e.g., behavior setting). As the population of online students becomes more diverse (Lee, 2017), future research could examine where younger students, who may have a higher need for peer-interaction and less control over their residence than working adult graduate students, carry out their online learning activities. Whether the learner is taking one course, or an entire online program may also influence what behavior setting or settings they study in, how long they stay in each, and how frequently they change settings.

Overall, the model has additional implications for pedagogical design and for students. The constant accessibility to computer and mobile devices has led to information overload, increasing demand on our attention, and facilitated multi-tasking both within the virtual environment and between the virtual and the physical environment (Terras and Ramsay, 2012; Stokols, 2018; Gaines, 2019). Research has begun to examine the effects of multitasking and associated division of attention on learning and learners’ coping strategies (cf. Levine et al., 2012; review by Chen and Yan, 2016). It seems likely that such factors as learner characteristics, learner motivation, task characteristics, and perception of relative importance of the tasks are important in influencing a learner’s ability to multi-task while learning (Coens et al., 2011; Gaines, 2019).

Concerns have been raised about how advances in information technology have encouraged browsing with shorter attention spans rather than in-depth reflection (Gaines, 2019). Future research may explore the temporal dimension of online learning, for example, how fragmentation of learning activities affects learning satisfaction and outcomes. For example, Seneca (2014) suggests designing apps downloadable in short bursts for quick access on mobile devices. However, there is some evidence that adult distance students preferred to set aside dedicated time for their academic studies (Lee and Chan, 2007). Thus, learning designers should consider whether online learning tasks should be designed for focused attention and active engagement in learners, or divided across several tasks to accommodate lifestyle integration (Lee and Chan, 2007). Designing for absorption and engagement will need to consider the management of interruptions (Terras and Ramsay, 2012).

Educators may consider how learning goals can be accomplished in different physical environments by incorporating various communication channels, synchronicities, and sensory modalities. For example, course materials presented in visual format and activities performed by hand can be learned in a relatively noisy physical environment. Audio content may be suitable when in poor lighting environments. Future research might explore how multimedia learning (Mayer, 2005) might be influenced by the physical environment in which the learner is located.

On the social side, educators need to design an online learning environment that fosters and enables social connections and social support. Providing institutional support for students (e.g., technical training and support) is crucial, considering that students may be using different devices and across different physical settings. Krull and Duart (2017) suggest further studies are required to examine what devices students use and how they access content and university services, perhaps with the use of learning analytics.

For students, they need to be aware of the effects that the physical and the virtual environment have on their learning and studying and be able to choose, set up, or control their physical environments for optimal learning effectiveness. Universities could provide information to help students achieve this objective. Research is needed to study strategies that would help learners with different learner characteristics succeed in online learning across multiple settings, such as learner autonomy, self-direction, and self-regulation (Grant, 2019).

## Author Contributions

The author confirms being the sole contributor of this work and has approved it for publication.

## Funding

The author has received no funding from any granting agencies for the writing of this manuscript. Athabasca University provides funds to cover the open access publication fees.

## Conflict of Interest

The author declares that the research was conducted in the absence of any commercial or financial relationships that could be construed as a potential conflict of interest.

## Publisher’s Note

All claims expressed in this article are solely those of the authors and do not necessarily represent those of their affiliated organizations, or those of the publisher, the editors and the reviewers. Any product that may be evaluated in this article, or claim that may be made by its manufacturer, is not guaranteed or endorsed by the publisher.
